# Comparison of HPV type distribution in high-grade cervical lesions and cervical cancer: a meta-analysis

**DOI:** 10.1038/sj.bjc.6601024

**Published:** 2003-07-01

**Authors:** G M Clifford, J S Smith, T Aguado, S Franceschi

**Affiliations:** 1 Unit of Field and Intervention Studies, International Agency for Research on Cancer, 150, cours Albert Thomas, 69008, Lyon, France; 2 Department of Vaccines and Biologicals, WHO, Geneva, Switzerland

**Keywords:** Human papillomavirus, high grade intraepithelial lesions, cervical cancer, squamous cell carcinoma, epidemiology, meta-analysis

## Abstract

Particular types of human papillomavirus (HPV) infection may preferentially progress from high-grade squamous intraepithelial lesions (HSIL) to squamous cell carcinoma of the cervix (SCC). We performed a meta-analysis of published data to compare HPV type distribution in HSIL and SCC. HPV16, 18 and 45 were each more prevalent in SCC than HSIL, whereas the reverse was true for other oncogenic types including HPV31, 33, 52 and 58. These data suggest that HSILs infected with HPV16, 18 and 45 preferentially progress to SCC. This may have implications for follow-up protocols of future HPV-based cervical cancer screening programmes and for HPV vaccine trials.

Epidemiological studies have established human papillomavirus (HPV) infection as the central cause of invasive cervical cancer (ICC) and its precursor lesions (Walboomers *et al*, 1999). However, only a fraction of precancerous lesions progress to ICC. A strong candidate factor for differential progression is HPV type (Lorincz *et al*, 1992).

Identifying HPV types that preferentially progress from high-grade squamous intraepithelial lesions (HSIL) to ICC has implications not only for follow-up protocols in ICC screening programmes, but also for prophylactic type-specific HPV vaccine trials. For ethical reasons, final outcome measures in such trials will be the prevention of HSIL. However, it is important to know whether the HPV type distribution in HSIL is representative of those that go on to cause cancer.

Articles presenting HPV type-specific prevalence data were identified from *Medline*. Studies had to include at least 20 cases of squamous cell or histologically unspecified cervical cancer (Clifford *et al*, 2002) and/or 20 histologically verified cases of HSIL. In this study, HSIL refers both to lesions classified by the Bethesda system, that is, CIN2/3, and those classified separately as CIN2 and CIN3. Studies had to use polymerase chain reaction (PCR)-based assays to identify HPV, and to present prevalence of at least one type other than HPV6, 11, 16 or 18 (Clifford *et al*, 2002).

This report includes 8594 squamous cell carcinoma of the cervix (SCC) cases (including 2725 of unspecified histology), as previously reported in Clifford *et al* (2002), and 4338 HSIL cases (1733 reported as CIN2/3, 1824 as CIN3, 729 as CIN2 and 52 as cervical carcinoma *in situ*)(detailed information on the HSIL studies is reported in the Appendix). Compared to SCC, cases of HSIL were more likely to be from (i) Europe and South/Central America rather than other regions, (ii) studies that detected HPV from exfoliated cells rather than biopsy specimens and (iii) studies that used ‘broad’-spectrum (MY09/11, GP5+/6+ and SPF10) rather than other PCR primers (Table 1

**Table 1:** Distribution of SCC and HSIL cases by region and study characteristics

**Lesion**	**No. of studies**	**No. of cases**	**Source region (% of cases)**	**Cervical specimen for HPV testing (% of cases)**	**PCR primers used (% of cases)**
SCC	78	8594	Africa (6.9), Asia (31.7),		Broad spectrum^a^ (61.9)
			Europe (32.0), North America/Australia	Biopsies (83.4)	Narrow spectrum^b^ (15.5)
			(13.0), South/Central America (16.5)	Exfoliated cells (16.6)	Combination/other (16.3)
					Type-specific only (6.4)
					
					Broad spectrum^a^ (80.8)
HSIL	53	4338	Africa (1.8), Asia (16.7), Europe	Biopsies (34.1)	Narrow spectrum^b^ (7.9)
			(52.4), North America (10.3),	Exfoliated cells (65.9)	Combination/other (7.4)
			South/Central America (18.8)		Type-specific only (3.9)

HPV=human papillomavirus; SCC=squamous cell/unspecified carcinoma of the cervix; HSIL=high-grade squamous intraepithelial lesion; PCR=polymerase chain reaction;

a ‘Broad’-spectrum PCR primers include MY09/11, GP5+/6+ and SPF10.

b ‘Narrow’-spectrum PCR primers include GP5/6, L1C1/C2 and PU1M/2R.

).

Type-specific prevalence is presented for the 14 most common high-risk (HR) types identified in SCC (Table 2

**Table 2:** Comparison of overall and type-specific HPV prevalence between SCC and HSIL cases

	**SCC**		**HSIL**		**SCC : HSIL**	
**HPV type**	*n*	**HPV (%)**	*n*	**HPV (%)**	**prevalence ratio^a^**	
All	8550	87.6	4338	84.2	1.04	(1.03, 1.06)
						
16	8594	54.3	4338	45.0	1.21	(1.16, 1.26)
18	8502	12.6	4338	7.1	1.79	(1.56, 2.10)
33	8449	4.3	4302	7.2	0.59	(0.53, 0.68)
45	5174	4.2	2214	2.3	1.85	(1.35, 2.91)
31	7204	4.2	4036	8.8	0.48	(0.43, 0.54)
58	5646	3.0	2175	6.9	0.43	(0.37, 0.52)
52	5304	2.5	2153	5.2	0.48	(0.40, 0.60)
35	6223	1.0	2690	4.4	0.22	(0.18, 0.27)
59	4488	0.8	1636	1.5	0.55	(0.38, 0.97)
56	4493	0.7	2110	3.0	0.23	(0.18, 0.31)
51	4580	0.6	2171	2.9	0.20	(0.16, 0.27)
68	4148	0.5	1763	1.0	0.50	(0.33, 1.04)
39	3899	0.4	1841	1.1	0.35	(0.24, 0.66)
66	4799	0.2	1778	2.1	0.10	(0.08, 0.15)

HPV=human papillomavirus; SCC=squamous cell/unspecified carcinoma of the cervix; HSIL=high-grade squamous intraepithelial lesion.

a With 95% confidence intervals.

). As not all studies tested for all 14 types, sample size varies between type-specific analyses. Type-specific prevalence is expressed as a percentage of all cases tested for HPV, and thus represents the prevalence in either single or multiple infections.

Overall, HPV prevalence was slightly higher in SCC cases (87.6%) than HSIL (84.2%) (SCC : HSIL ratio 1.04, 95% CI 1.03–1.06) (Table 2). HPV16 was the most common type in both SCC (54.3%) and HSIL (45.0%), but was more prevalent in SCC (ratio of 1.21, 95% CI 1.16–1.26). HPV18 was also more prevalent in SCC (12.6%) than in HSIL (7.0%), with a ratio of 1.79 (95% CI 1.56–2.10). HPV45 was associated with a ratio of 1.85 (95% CI 1.35–2.91), similar to that of HPV18. All other HR types included in the analysis had ratios between 0.1 and 0.6 (Table 2).

The SCC : HSIL ratios for the eight most common HPV types were additionally calculated within more homogeneous study subgroups: (i) studies that did not report any multiple infections (6558 SCC, 2182 HSIL), (ii) studies testing for HPV from biopsies (7128 SCC, 1483 HSIL) and (iii) studies using ‘broad’-spectrum PCR primers (5318 SCC, 3502 HSIL). The SCC : HSIL ratios were also calculated separately for HSILs classified by the Bethesda system and for CIN3 only. Across all these subanalyses, SCC : HSIL ratios remained consistent for HPV16 (range: 1.04–1.25), HPV18 (1.46–1.93) and HPV45 (1.20–4.61). HPV31, 33, 35, 52 and 58 were consistently associated with ratios of 0.3–0.9, with the exception of HPV58 for biopsy studies (1.06, 95% CI 0.73–2.08).

Where sample size permitted, subanalyses were also stratified by region. When estimated from studies within Asia, Europe and South/Central America, respectively, there was no material difference in SCC : HSIL ratios for HPV16 (1.46, 1.17, 1.40), HPV18 (1.74, 2.02, 1.46), HPV45 (4.35, 1.39, 1.20), HPV33 (0.56, 0.62, 0.76), HPV52 (0.39, 0.26, 0.64) or HPV58 (0.55, 0.24, 0.30). However, notably high ratios were observed for HPV31 in South/Central America (1.13, 95% CI 0.84–1.70) in comparison to Europe (0.41, 95% CI 0.36–0.48) and Asia (0.43, 95% CI 0.31–0.68), and for HPV58 in China (including Taiwan and Hong Kong) (1.27, 95% CI 0.85–2.51) in comparison to non-Chinese Asian countries (0.37, 95% CI 0.27–0.58), raising the possibility of localised variation in the malignant potential of particular HPV types (Chan *et al*, 2002).

Our findings suggest that worldwide, HSIL infected with HPV16, 18 or 45 are more likely to progress to SCC than HSIL infected with other HR types. This could be interpreted in two ways: either these types have a greater potential to induce fully malignant transformation, and/or these infections somehow preferentially evade the host immune system. Compared to other HPV types, HPV18 has been associated with increased oncogenic potential in cell culture, screening failures and poorer cancer prognosis (Hildesheim *et al*, 1999; Schwartz *et al*, 2001; Woodman *et al*, 2003). Thus, HPV18 enrichment in SCC may reflect its greater oncogenic potential. Given its genetic similarity to HPV18, this may also be true for HPV45. Conversely, compared to other HPV types, HPV16 infections are more likely to persist and progress to HSIL (Molano *et al*, in press). Both persistence of infection and progression to HSIL have been shown associated with HPV16 variants (Londesborough *et al*, 1996). Thus, HPV16 enrichment in SCC may be related to its greater ability to escape immune surveillance compared to other types.

Even in countries with established screening programmes, women still die from rapidly progressing cancers that escape periodic examination. Given that HPV16, 18 and 45 appear to have greater progressive potential, and in the event that future cervical screening programmes include HPV typing, women infected with HPV16, 18 and 45 may require closer surveillance than women infected with other HR HPV types.

The demonstration that the HPV type distribution in HSIL is not entirely representative of those that go on to cause cancer also has important implications for prophylactic type-specific HPV vaccine evaluation. This is because any beneficial effect identified by randomised trials from the proportion of HSIL preventable by HPV16 or HPV16/18 vaccines may be an underestimate of the beneficial effect on the prevention of ICC.

## Appendix

Study methods and type-specific prevalence of human papillomavirus by study and by region are summarised in Table A1

**Table A1:**

							**HPV-specific prevalence (% of all cases tested)**														
**First author**	**Reference**	**Country**	**HPV DNA source**	**PCR primers used to identify all HPV +ve**	**No. cases**	**CINII/CINIII/CIS/HSIL**	**Any**	**16**	**18**	**45**	**31**	**33**	**58**	**52**	**35**	**59**	**56**	**51**	**68**	**39**	**66**
*Africa*																					
La Ruche	*Int J Cancer* (1998)	Ivory Coast	Exfol. cells	MY09/11	49	0/0/0/49	77.6	30.6	10.2	0.0	6.1	8.2	8.2	4.1	0.0	0.0	4.1	0.0	2.0	0.0	
de Vuyst	*Sex Transm Dis* (2003)	Kenya	Exfol. cells	SPF10	29	0/0/0/29	96.6	34.5	3.4	6.9	6.9	3.4	6.9	24.1	17.2	0.0	3.4	10.3	6.9	0.0	10.3
																					
*Africa sub-total*					78	0/0/0/78	84.6	32.1	7.7	2.6	6.4	6.4	7.7	11.5	6.4	0.0	3.8	3.8	3.8	0.0	10.3
																					
*Asia*																					
Chan MKM	*Gynecol Oncol* (1996)	China	Exfol. cells	MY09/11	45	10/35/0/0	55.6	24.4	8.9		0.0	4.4									
Chan PKS	*J Med Virol* (1999)	China	Exfol. cells	MY09/11	89	29/60/0/0	58.4	25.8	4.5	0.0	3.4	6.7	11.2	1.1	0.0	0.0	0.0	0.0	0.0	1.1	0.0
Wu CH	*Sex Transm Dis* (1994)	China	Fixed biopsies	TS-PCR only	34	13/15/6/0	76.5	35.3	20.6		0.0	5.9									
Nagai Y	*Gynecol Oncol* (2000)	Japan	Exfol. cells	L1C1/L1C2	58	0/58/0/0	96.6	37.9	3.4		8.6	15.5	6.9		1.7						
Saito J	*Jpn J Obstet Gynecol Pract* (2001)	Japan	Exfol. cells	L1C1/L1C2	38	0/0/0/38	100.0	34.2	18.4	0.0	7.9	7.9	13.2	34.2	0.0	15.8	0.0	13.2	2.6	0.0	2.6
Sasagawa T	*Cancer Epidemiol Biomarkers Prev* (2001)	Japan	Exfol. cells	LCR-E7	137	0/0/0/137	91.2	35.8	2.2	2.2	9.5	2.2	13.1	10.9	2.9	0.0	5.8	7.3	0.0	0.0	0.7
Yoshikawa H	*Jpn J Cancer Res* (1991)	Japan	Biopsies	L1C1/L1C2	31	0/0/20/11	90.3	38.7	9.7		6.5	12.9	3.2	12.9							
Oh YL	*Cytopathology* (2001)	Korea	Exfol. cells	pU-1M/pU-2R	42	0/0/0/42	73.8	40.5	7.1		7.1	19.0									
Lai HC	*Int J Cancer* (2003)	Taiwan	Exfol. cells	MY09/11	141	0/0/0/141	63.8	25.5	1.4			4.3	9.9	11.3							
Ekalaksananan T	*J Obstet Gynaecol. Res* (2001)	Thailand	Exfol. cells	E1 primers	40	10/4/26/0	65.0	7.5	15.0			2.5									
Lertworapreecha M	*Southeast Asian J Trop. Med Public Health* (1998)	Thailand	Fixed biopsies	MY09/11	50	0/50/0/0	74.0	36.0	12.0			8.0									
Limbaiboon T	*Southeast Asian J Trop. Med Public Health* (2000)	Thailand	Fixed biopsies	MY09/11	21	0/21/0/0	100.0	33.3	14.3			4.8									
																					
*Asia sub-total*					726	62/243/52/369	76.4	31.4	6.9	1.0	4.9	6.7	10.5	11.2	1.6	2.3	3.0	4.0	0.4	0.5	0.7
																					
																					
*Europe*																					
Baay MFD	*Eur J Gynaecol. Oncol* (2001)	Belgium	Fixed biopsies	GP5+/6+	97	42/55/0/0	82.5	56.7	6.2	0.0	0.0	6.2	6.2	2.1	2.1	0.0	2.1	1.0	1.0	0.0	1.0
Tachezy R	*Hum. Genet.* (1999)	Czech Republic	Exfol. cells	MY09/11	88	0/0/0/88	58.0	43.2	5.7	3.4	1.1	6.8	0.0	1.1	0.0	0.0	0.0	1.1	0.0	1.1	0.0
Sebbelov	*Res Virol.* (1994)	Denmark	Fixed biopsies	GP5/6	34	0/34/0/0	91.2	85.3	0.0		0.0	29.4									
Bergeron B	*Am J Surg Pathol* (1992)	France	Fresh biopsies	L1 primers	53	0/0/0/53	92.5	56.6	3.8			1.9									
Merkelbach-Bruse S	*Diagn Mol. Pathol* (1999)	Germany	Fixed biopsies	GP5/6	88	21/67/0/0	78.4	61.4	1.1		3.4	1.1									
Meyer T	*Int J Gynecol Cancer* (2001)	Germany	Fresh biopsies	MY09/11	288	0/0/0/288	94.4	46.2	6.6	1.4	13.2	9.4	1.7	5.6	3.1	0.7	1.4	1.0	0.3	1.4	2.1
Nindl I	*J Clin Pathol* (1999)	Germany	Exfol. cells	GP5+/6+	65	31/34/0/0	87.7	56.9	6.2	1.5	18.5	7.7	0.0	0.0	0.0	0.0	0.0	0.0	0.0	0.0	1.5
Nindl I	*Int J Gynecol Pathol* (1997)	Germany	Exfol. Cells	GP5+/6+	85	0/0/0/85	83.5	36.5	2.4		5.9	12.9									
Labropoulou V	*Sex Transm Dis* (1997)	Greece	Fresh biopsies	MY09/11	50	0/0/0/50	88.0	36.0	12.0		6.0	6.0						4.0			0.0
Paraskevaidis E	*Gynecol Oncol* (2001)	Greece	Exfol. cells	MY09/11	28	0/0/0/28	89.3	35.7	7.1	3.6	25.0	14.3	0.0	0.0	0.0		0.0	0.0		0.0	
Sebbelov	*Res Virol*. (1994)	Greenland	Fixed biopsies	GP5/6	30	0/30/0/0	63.3	70.0	3.3		6.7	10.0									
Butler D	*J Pathol* (2000)	Ireland	Fixed biopsies	TS-PCR only	27	0/27/0/0	85.2	70.4	3.7		3.7	3.7	0.0	0.0							
O'Leary JJ	*Hum. Pathol* (1998)	Ireland	Fixed biopsies	GP5/6	20	0/20/0/0	95.0	95.0	0.0			0.0									
Laconi	*Pathologica* (2000)	Italy	Fixed biopsies	GP5+/6+	36	19/17/0/0	100.0	50.0	8.3		2.8			2.8	5.6		5.6	2.8	0.0	0.0	
Zerbini M	*J Clin Pathol* (2001)	Italy	Exfol. cells	MY09/11	89	0/0/0/89	79.8	50.6	3.4	2.2	7.9	9.0									
Medeiros R	*Proceedings of International Meeting of Gynaecological Oncology* (1997)	Portugal	Fixed biopsies	MY09/11	78	10/68/0/0	85.9	82.1	0.0			1.3									
Bosch	*Cancer Epidemiol Biomarkers Prev* (1993)	Spain	Exfol. cells	MY09/11	157	0/157/0/0	70.7	49.0	0.6		1.3	5.7			0.6						
Kalantari M	*Hum. Pathol* (1997)	Sweden	Exfol. cells	MY09/11	164	69/95/0/0	82.9	36.0	7.3		7.3	10.4									
Zehbe I	*Virchows Arch* (1996)	Sweden	Fixed biopsies	GP5+/6+	103	55/48/0/0	95.1	50.5	9.7	1.9	7.8	9.7	1.9	0.0	7.8		1.9	0.0			
Bollen LJM	*Am J Obstet Gynecol* (1997)	The Netherlands	Exfol. cells	CpI/CPIIG	91	24/64/0/3	97.8	36.3	4.4	4.4	18.7	5.5	7.7	2.2	4.4		1.1	4.4	3.3		
Cornelissen MTE	*Virchows Arch B Cell Pathol Incl. Mol. Pathol* (1992)	The Netherlands	Fixed biopsies	MY09/11	89	16/73/0/0	88.8	52.8	6.7		12.4	5.6									
Reesink-Peters N	*Eur J Obstet Gynecol Reprod Biol* (2001)	The Netherlands	Exfol. cells	SPF10	216	44/172/0/0	97.7	56.9	13.9		19.4	11.6					8.3				
Arends MJ	*Hum. Pathol* (1993)	UK	Fixed biopsies	TS-PCR only	40	20/20/0/0	60.0	50.0	10.0			0.0									
Cuzick J	*Br J Cancer* (1994)	UK	Exfol. cells	TS-PCR only	73	12/61/0/0	91.8	63.0	20.5		26.0	16.4			2.7						
Giannoudis A	*Int J Cancer* (1999)	UK	Fixed biopsies	GP5+/6+	118	31/87/0/0	100.0	68.6	4.2	0.0	14.4	11.0	3.4	0.8	2.5	0.0	0.0	2.5	0.0	0.8	2.5
Herrington CS	*Br J Cancer* (1995)	UK	Exfol. cells	MY09/11	38	12/26/0/0	92.1	50.0	7.9		18.4	7.9									
Southern SA	*Diagn Mol. Pathol* (1998)	UK	Fixed biopsies	GP5+/6+	26	0/26/0/0	100.0	61.5	7.7	0.0	15.4	3.8	0.0	0.0	0.0	0.0	0.0	0.0	0.0	0.0	3.8
																					
*Europe sub-total*					2271	406/1181/0/684	87.1	52.6	6.5	1.7	10.5	8.4	2.6	2.4	2.6	0.3	2.5	1.5	0.6	0.8	1.6
																					
*North America*																					
Sellors JW	*Can Med Assoc J*. (2000)	Canada	Exfol. cells	MY09/11	58	0/0/0/58	98.3	75.9	8.6	0.0	27.6	5.2	0.0	0.0	0.0	0.0	0.0	0.0	0.0	0.0	0.0
Adam E	*Am J Obstet Gynecol* (1998)	USA	Exfol. cells	MY09/11	257	0/0/0/257	78.2	51.0	13.6		1.9	4.7			13.6						
Aoyama C	*Diagn Mol. Pathol* (1998)	USA	Fixed biopsies	MY09/11	21	4/15/0/2	95.2	52.4	0.0		19.0	19.0									
Schiff M	*Am J Epidemiol* (2000)	USA	Exfol. cells	MY09/11	112	70/42/0/0	77.7	17.0	4.5	1.8	22.3	4.5	16.1	4.5	4.5	4.5	12.5	4.5	2.7	6.3	9.8
																					
*North America sub-total*					448	74/57/0/317	81.5	45.8	10.0	1.2	11.2	5.4	10.6	2.9	9.4	2.9	8.2	2.9	1.8	4.1	6.5
																					
*South/Central America*																					
Abba MC	*International Papillomavirus Conference Proceedings* (2001)	Argentina	Fixed biopsies	MY09/11	86	13/24/0/49	97.7	50.0	14.0		7.0	2.3						7.0			
Alonio LV	*Medicina (B Aires)* (2000)	Argentina	Biopsies	GP5+/6+	36	0/36/0/0	80.6	41.7	11.1		0.0	5.6									
Lorenzato F	*Int J Gynecol Cancer* (2000)	Brazil	Exfol. cells	MY09/11	60	0/0/0/60	86.7	56.7	3.3	3.3	3.3	8.3	10.0	0.0	1.7						
Bosch	*Cancer Epidemiol Biomarkers Prev* (1993)	Colombia	Exfol. cells	MY09/11	125	0/125/0/0	63.2	32.8	0.0		2.4	2.4			1.6						
Herrero R	*J Natl Cancer Inst* (2000)	Costa Rica	Exfol. cells	MY09/11	125	0/0/0/125	88.8	44.8	5.6	2.4	6.4	3.2	9.6	7.2	3.2	0.8	3.2	7.2	0.8	3.2	0.0
Ferrera A	*Int J Cancer* (1999)	Honduras	Exfol. cells	MY09/11	83	36/47/0/0	80.7	34.9	7.2	3.6	8.4	4.8	7.2	1.2	1.2	0.0	1.2	0.0	0.0	0.0	0.0
Rattray	*J Infect. Dis* (1996)	Jamaica	Exfol. cells	MY09/11	66	27/39/0/0	80.3	24.2	4.5	13.6	9.1	7.6			13.6						
Strickler HD	*J Med Virol.* (1999)	Jamaica	Exfol. cells	MY09/11	183	111/72/0/0	92.3	23.5	10.9	4.4	8.7	7.7	12.6	9.3	11.5	5.5	2.2	4.9	2.2	1.1	4.9
Illades-Aguiar	*International Papillomavirus Conference Proceedings* (2001)	Mexico	Fresh biopsies	MY09/11	27	0/0/0/27	85.2	37.0	3.7	0.0	14.8	14.8	3.7	0.0	0.0	0.0	0.0	0.0	0.0	0.0	0.0
Torroella-Kouri M	*Gynecol Oncol* (1998)	Mexico	Exfol. cells	MY09/11	24	0/0/0/24	83.3	58.3	12.5	0.0	0.0	8.3	12.5	0.0	0.0	0.0	0.0	4.2	0.0	0.0	0.0
																					
*South/Central America sub-total*					815	187/343/0/285	84.3	36.9	7.1	4.4	6.4	5.5	10.2	5.4	5.5	2.5	2.0	4.7	1.1	1.4	2.0
																					
Total					4338	729/1824/52/1733	84.2	45.0	7.1	2.3	8.8	7.2	6.9	5.2	4.4	1.5	3.0	2.9	1.0	1.1	2.1

Exfol. Cells=exfoliated cells.
